# Comparison between the efficiency of pharmacotherapy and cognitive behavioral therapy in reducing captagon (fenethylline) dependence and relapse prevention

**DOI:** 10.1192/bjo.2021.454

**Published:** 2021-06-18

**Authors:** Mohammed Binnwejim, Atheer Alhumade, Deiaaeldin Hosny, Mohamed Alhabib

**Affiliations:** 1 Imam Muhammed Bin Saud Islamic University; 2 Clinical Psychology and Psychotherapy

## Abstract

**Aims:**

To examine the therapeutic efficacy and effectiveness of cognitive behavior therapy and pharmacotherapy in the treatment of Major Captagon (Fenethylline) Dependence.

**Method:**

A 41 outpatients males selected for the study, diagnosed as they are suffering from Captagon Dependence according to the DSM-5, with mean age 34.58 ± 5.11. The sample was divided into three experimental groups, (A) (N = 14) treated by cognitive behavior therapy (CBT) and pharmacotherapy in combination. (B) (N = 13) treated by CBT alone. (C) (N = 14) treated by pharmacotherapy alone. All groups were assigned to four measurements, one for the baseline before any treatment interventions, one post-treatment evaluation and two for follow-up within a short and long time. Non-parametric statistics were used to analyze the data collected by SPSS.

**Result:**

There is no significant intra-group differences were found in terms of baseline assessment. There was no significant discrepancy between the first and the second group except in the term of reducing Captagon craving, as it was clearer in the first group in comparison with other groups. There was a clear significant discrepancy between the first and third groups, for all the study variables and it is phases of assessment especially follow-up. There was a clear degree of differences among the second and the third group, through the different phases of post-assessment, which refers to the great efficacy and effectiveness of CBT in Treating Captagon Dependence CBT was proved to be more effective than pharmacotherapy in the treatment of Captagon Dependence. The combination of CBT and pharmacotherapy was more effective than each other alone in the treatment of Captagon Dependence and Relapse Prevention.

**Conclusion:**

Available evidence suggests that cognitive–behavioral therapy is an effective intervention method for psychological aspects of automatic thoughts, depression, negative health beliefs, craving, and relapse prevention, although its efficacy in reducing Captagon (Fenethylline) dependence.

